# Brain iron deposition after Ferumoxytol-enhanced MRI: A study of Porcine Brains

**DOI:** 10.7150/ntno.46356

**Published:** 2020-06-18

**Authors:** Ashok Joseph Theruvath, Maryam Aghighi, Michael Iv, Hossein Nejadnik, Jonathan Lavezo, Laura Jean Pisani, Heike Elisabeth Daldrup-Link

**Affiliations:** 1 Department of Radiology, Molecular Imaging Program at Stanford, Stanford University, CA, USA.; 2 Department of Diagnostic and Interventional Radiology, University Medical Center of the Johannes Gutenberg-University Mainz, 55131 Mainz, Germany.; 3 Department of Pathology, School of Medicine, Stanford University, Stanford, CA, USA.

**Keywords:** iron oxide nanoparticle, brain deposition, MRI

## Abstract

Recent evidence of gadolinium deposition in the brain has raised safety concerns. Iron oxide nanoparticles are re-emerging as promising alternative MR contrast agents, because the iron core can be metabolized. However, long-term follow up studies of the brain after intravenous iron oxide administration have not been reported thus far. In this study, we investigated, if intravenously administered ferumoxytol nanoparticles are deposited in porcine brains.

**Methods:** In an animal care and use committee-approved prospective case-control study, ten Göttingen minipigs received either intravenous ferumoxytol injections at a dose of 5 mg Fe/kg (n=4) or remained untreated (n=6). Nine to twelve months later, pigs were sacrificed and the brains of all pigs underwent *ex vivo* MRI at 7T with T2 and T2*-weighted sequences. MRI scans were evaluated by measuring R2* values (R2*=1000/T2*) of the bilateral caudate nucleus, lentiform nucleus, thalamus, dentate nucleus, and choroid plexus. Pig brains were sectioned and stained with Prussian blue and evaluated for iron deposition using a semiquantitative scoring system. Data of ferumoxytol exposed and unexposed groups were compared with an unpaired t-test and a Mann-Whitney U test.

**Results:** T2 and T2* signal of the different brain regions was not visually different between ferumoxytol exposed and unexposed controls. There were no significant differences in R2* values of the different brain regions in the ferumoxytol exposed group compared to controls (p>0.05). Prussian blue stains of the same brain regions, scored according to a semiquantitative score, were not significantly different either between the ferumoxytol exposed group and unexposed controls (p>0.05).

**Conclusions:** Our study shows that intravenous ferumoxytol doses of 5-10 mg Fe/kg do not lead to iron deposition in the brain of pigs. We suggest iron oxide nanoparticles as a promising alternative for gadolinium-enhanced MRI.

## Introduction

Gadolinium-based contrast agents (GBCAs) are widely used for clinical magnetic resonance imaging (MRI). However, classical GBCAs have raised safety concerns due to a risk of gadolinium deposition in the brain 1. Iron oxide nanoparticles are re-emerging as a promising alternative to GBCAs. Ferumoxytol, a food and drug administration (FDA)-approved iron supplement, is immediately available as an MRI contrast agent through “off-label” use. The large size of ferumoxytol nanoparticles leads to limited extravasation in normal tissues, including the brain 2. Various authors used ferumoxytol for clinical MRI of the cardiovascular system, brain pathologies and body imaging, among others 2. While previous investigators reported absent ferumoxytol enhancement in the normal brain of patients on MRI 2, long-term follow up studies have not been performed. Our team conducts ferumoxytol-MRI of large animals, which provided a unique opportunity to obtain imaging-histopathological correlations. The purpose of our study was to evaluate, if intravenous ferumoxytol administration leads to long-term iron deposition in brain tissue.

## Methods

Studies have been approved by the animal care and use committee of our institution. We prospectively investigated the brains of 10 Göttingen minipigs: Four pigs received one (n=3) or two (n=1) intravenous injection(s) of ferumoxytol at a dose of 5 mg Fe/kg and were sacrificed 9-12 months later for *ex vivo* brain MRI. Six pigs were not injected with ferumoxytol and served as untreated controls. Board-certified veterinarians placed an intravenous line into the ear vein and checked the venous access through a saline flush before ferumoxytol injection. The same venous access was used to anesthetize the pig. To validate that ferumoxytol nanoparticles had been successfully injected intravenously, MRI scans of the knee joint for ferumoxytol treated pigs showed negative (hypointense) contrast enhancement within the bone marrow and no contrast enhancement in the bone marrow of control pigs **(Figure S1)**.

The brains of all pigs underwent *ex vivo* MRI on a 7T MRI scanner (Bruker Biospin, Billerica, MA) using T2 weighted fast spin echo sequences (TR/TE/α =4952 ms/75 ms/90º, SL=1 mm) and T2* weighted gradient echo sequences (1366 ms/3.5-48.5 ms/70º, SL=1 mm) for creation of T2* maps. One investigator (AJT) measured R2* values (R2*=1000/T2*) of the bilateral caudate nucleus, lentiform nucleus, thalamus, dentate nucleus, and choroid plexus by carefully placing operator-defined regions of interest (ROIs) in the specific brain areas on T2* maps. R2* is proportional to tissue iron concentration 3. We focused on brain areas where extravasation was previously described for other contrast agents 4. Brain specimens were cut, stained with Prussian blue and evaluated for iron deposition by a neuropathology fellow (JL) and one radiology resident (AJT), using a semiquantitative scoring system (1= no iron, 2= focal iron, 3= patchy iron, 4= diffuse iron). To validate our histopathological staining method, we added a positive control of a ferumoxytol exposed liver parenchyma. Analysis of T2* maps and pathology was performed with blinding to the experimental groups. R2* data and histology score were compared between ferumoxytol exposed brains and controls, using an unpaired t-test and a Mann-Whitney U test, respectively (p<0.05).

## Results

**Figure 1:**
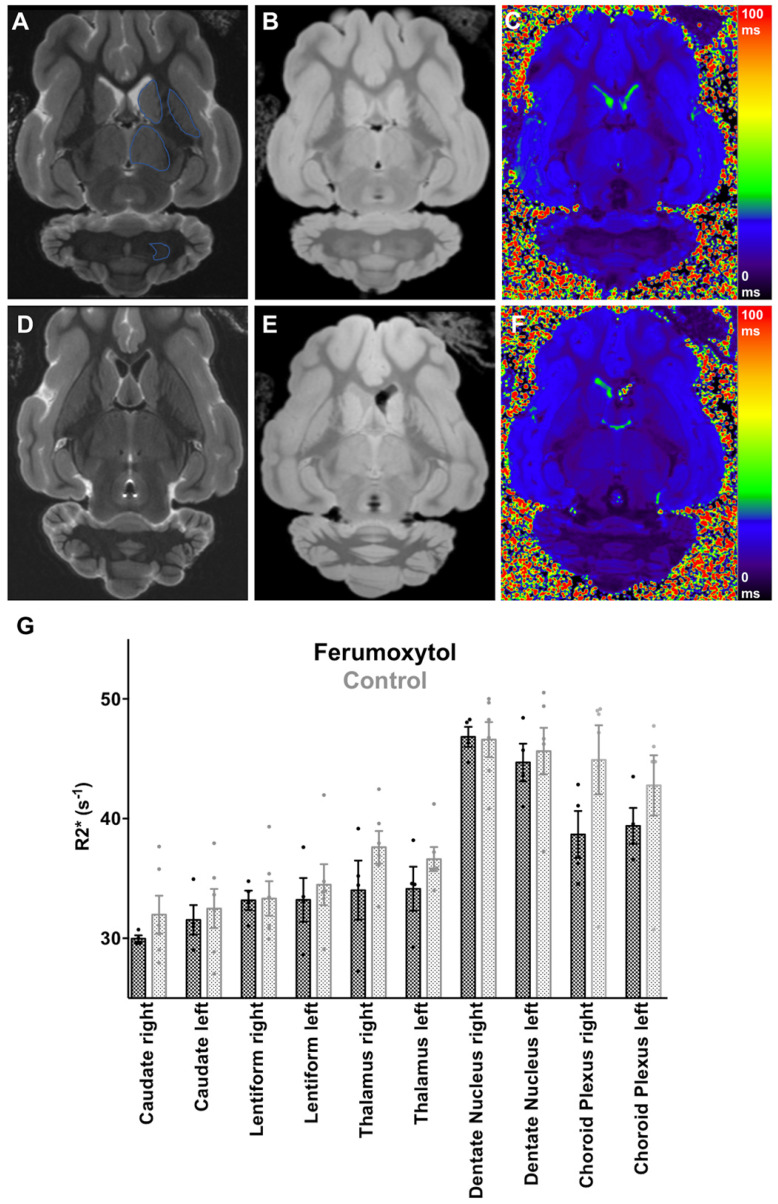
*Ex vivo* Brain MRI with R2* Quantification of Specific Brain Areas. **(A)** T2-weighted (TR/TE/α=4952/75/90) and **(B)** T2*-weighted (TR/TE/α=1366/3.5/70) image with **(C)** corresponding color encoded T2* map of a ferumoxytol exposed pig brain with regions of interest (blue line) in the caudate nucleus, lentiform nucleus, thalamus and dentate nucleus. **(D)** T2-weighted and **(E)** T2*-weighted image with **(F)** corresponding color encoded T2* map of a control pig brain. No visual difference between the two groups is demonstrated. **(G)** Scatterplot with bar shows corresponding R2* values with no significant differences for any of the quantified brain regions between ferumoxytol exposed and control pig brains (p>0.05). Data are means and standard errors of the mean of four ferumoxytol-exposed pigs and six controls.

**Figure 2:**
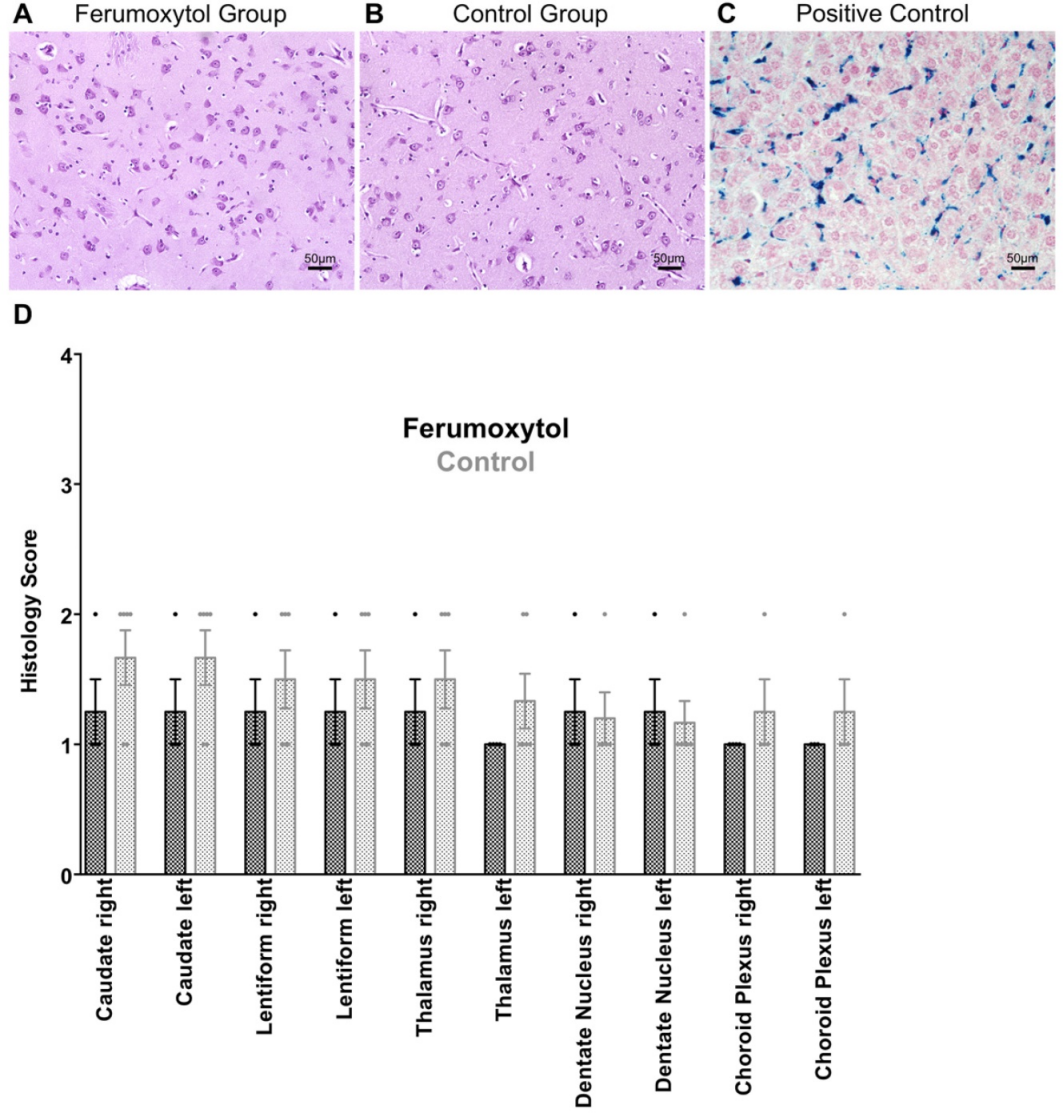
Prussian blue staining of specific brain areas. **(A)** Prussian blue staining of ferumoxytol exposed caudate nucleus and **(B)** unexposed caudate nucleus shows no sign of iron oxide nanoparticle retention. **(C)** Positive control with Prussian blue staining of ferumoxytol exposed liver parenchyma. **(D)** Scatterplots with bars show no significant differences in histological scoring for iron deposition in different brain areas (p>0.05; 1= no iron, 2= focal iron, 3= patchy iron, 4= diffuse iron). Data are means and standard errors of the mean of four ferumoxytol-exposed pigs and six controls.

We did not note any visual difference in T2 or T2* signal of any brain area between ferumoxytol exposed animals and controls** (Figure 1A-F)**. Accordingly, there were no significant differences in R2* values of different brain regions in the ferumoxytol exposed group compared to controls, respectively (**Figure 1G**): right caudate nucleus (29.97±0.27 vs. 31.98±1.56; p=0.34) left caudate nucleus (31.54±1.24 vs. 32.49±1.64; p=0.69), right lentiform nucleus (33.18±0.81 vs. 33.32±1.45; p=0.94), left lentiform nucleus (33.21±1.83 vs. 34.49±1.71; p=0.63), right thalamus (34.03±2.48 vs. 37.62±1.36; p=0.20), left thalamus (34.14±1.84 vs. 36.63±1.01; p=0.23), right dentate nucleus (46.82±0.84 vs. 46.59±1.46; p=0.91), left dentate nucleus (44.68±1.57 vs. 45.64±1.94; p=0.73), right choroid plexus (38.68±1.96 vs. 44.91±2.88; p=0.15) and left choroid plexus (39.40±1.50 vs. 42.78±2.50; p=0.34). In addition, we did not find any difference in Prussian blue staining between the two groups (**Figure 2A-C**). The histology score in different brain regions was not significantly different in the ferumoxytol exposed group compared to controls (p>0.05; **Figure 2D**).

## Discussion

Our imaging-histopathological correlation suggests that ferumoxytol is not retained in brain tissue after intravenous administration at doses of 5-10 mg Fe/kg. Long-term follow up studies showed no significant differences in brain iron content between ferumoxytol exposed porcine brains and unexposed controls. This is in accordance with previous reports that described absence of iron enhancement of the normal brain within hours or days after ferumoxytol administration 2, 5.

Studies with GBCA demonstrated a correlation between cumulative GBCA dose and GBCA retention in the brain 1. Preclinical studies have highlighted the importance of the choroid plexus in the regulation of iron metabolism in the brain 6. Clinical studies have shown hypointense enhancement of the choroid plexus on MRI after multiple blood or iron transfusions 5, 7. Therefore, it is likely, that iron brain deposition is dose dependent.

Several studies determined the minimum quantity of iron oxide nanoparticles that can be detected. In a study using Molday iron oxide nanoparticles (diameter: 30 nm, similar size to ferumoxytol) the minimum detectable concentration in horse serum on a 7T MRI scanner was 38 pmol/l 8. Several clinical trials determined threshold iron concentrations that can be detected in tissues: In postmortem brain tissues from Alzheimer's disease patients, a threshold iron concentration of 50 µg Fe/g wet tissue was determined, above which iron could be detected in the brain on MR images at 4.7 Tesla 9. In another study of human postmortem brains specimen, iron concentration as low as 30 ± 12 µg Fe/g and up to 205 ± 32 µg Fe/g wet tissue correlated significantly with R2* values on a 3T MRI scanner 10. Studies comparing Prussian blue stains with MRI have reported an overlap of positive Prussian blue stains and T2^*^ hypointensities on MRI scans obtained on a 7T MRI scanner for patients with Parkinson's disease 11. In another study, hypointensities in the subthalamic nucleus on a 9.4T MRI corresponded with regions of high Prussian blue staining 12.

Our study used a 7T MRI scanner and iron-sensitive T2* sequences which can detect very low iron concentrations, as described in the literature. Therefore, differences in brain iron deposition between the groups in our study should have been detected with Prussian blue staining and MRI.

Previous clinical trials for MR imaging applications used doses of ferumoxytol from 1 to 8 mg Fe/kg 13-16. Our team uses a dose of 5 mg Fe/kg for clinical applications. This dose is comparable with previous studies and is lower than FDA-approved doses for treatment of anemia (2× 510mg per patient) 2, 17-20. In general, ferumoxytol nanoparticles are well tolerated and rare anaphylactic reactions have been reported in the adult patient population 21-24. This is in accordance with our experience in pediatric patients who received ferumoxytol for MRI and showed few and minor side effects 25. As recommended by the FDA, we diluted ferumoxytol 1:3 with saline and slowly infused it over 15 minutes to avoid complement activation-related pseudoallergies (CARPA) and hypotensive reactions, which are observed with rapid injections, and which can mimic true anaphylaxis 5.

Several investigators including our own group started to use ferumoxytol nanoparticles for MR imaging of vascular malformations 26, 27, cardiovascular abnormalities 28 and tumors in children 19. The sensitivity and specificity of ferumoxytol for imaging brain pathologies in patients has been extensively studied 29-32. Since ferumoxytol is FDA-approved for the treatment of anemia, results from this project can be directly translated to clinical applications via an off-label use. Therefore, ferumoxytol-enhanced MRI could address an important need for clinicians as a new alternative to Gd-enhanced MRI scans, which can pose a risk of long-term Gd-deposition in the brain of patients. The advantage of iron oxides compared to GBCA is that iron products can be metabolized. Of course, the capacity of the human body to metabolize iron products can be saturated with repetitive injections and the iron clearance can be impaired in case of metabolic diseases. Future studies will have to show, which iron doses and/or physiological conditions might lead to significant brain iron deposition or limited iron clearance.

## Supplementary Material

Supplementary figure S1.

## Abbreviations

GBCA: Gadolinium-based contrast agent
MRI: magnetic resonance imaging
FDA: food and drug administration
TR: time of repetition
TE: time of echo
SL: slice thickness
