# Supplementary material for: Brain iron deposition after Ferumoxytol-enhanced MRI: A study of Porcine Brains
Source: Nanotheranostics. 2020 Jun 18;4(4):195–200. doi: 10.7150/ntno.46356 (PMC7332795; doi:10.7150/ntno.46356)
Supplement: Supplementary file 1 — Supplementary figure S1. [file ntnov04p0195s1.pdf]

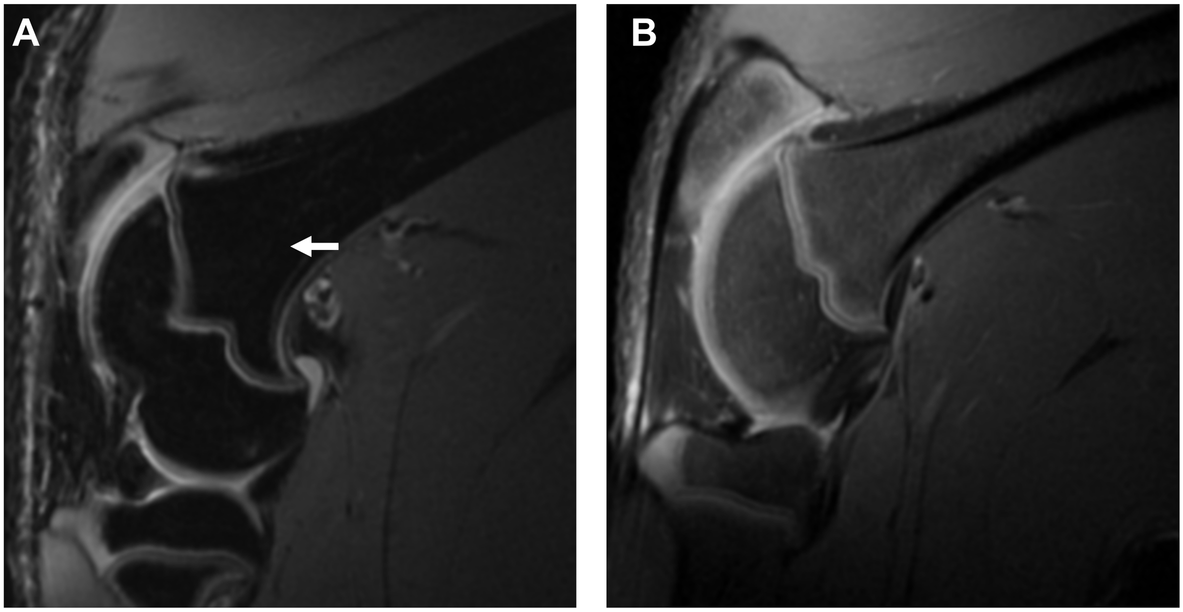

Figure S1: MRI scan of a porcine knee joint with and without intravenous ferumoxytol injection. **(A)** Sagittal proton density weighted image of the knee joint shows negative (hypointense) contrast enhancement in the bone marrow (arrow) after intravenous ferumoxytol injection. **(B)** Proton density weighted image of a knee joint without intravenous ferumoxytol injection demonstrates no contrast enhancement in the bone marrow.
